# Advances in Research on Antiviral Activities of Sulfated Polysaccharides from Seaweeds

**DOI:** 10.3390/ph15050581

**Published:** 2022-05-06

**Authors:** Qiang Wei, Guoqiang Fu, Ke Wang, Qiong Yang, Jiarui Zhao, Yuan Wang, Kai Ji, Shuliang Song

**Affiliations:** 1 Marine College, Shandong University, Weihai 264209, China; wq18086420598@163.com (Q.W.); wk1308866506@163.com (K.W.); yangqiong1237@163.com (Q.Y.); 201936684@mail.sdu.edu.cn (J.Z.); wangyuan1126@mail.sdu.edu.cn (Y.W.); 2 Weihaiwei People’s Hospital, Weihai 264200, China; fgq19681123@163.com; 3 Department of Plastic Surgery, China-Japan Friendship Hospital, Beijing 100029, China

**Keywords:** sulfated polysaccharides, seaweed, algae, antiviral, viruses, SARS-CoV-2, COVID-19

## Abstract

In recent years, various viral diseases have suddenly erupted, resulting in widespread infection and death. A variety of biological activities from marine natural products have gradually attracted the attention of people. Seaweeds have a wide range of sources, huge output, and high economic benefits. This is very promising in the pharmaceutical industry. In particular, sulfated polysaccharides derived from seaweeds, considered a potential source of bioactive compounds for drug development, have shown antiviral activity against a broad spectrum of viruses, mainly including common DNA viruses and RNA viruses. In addition, sulfated polysaccharides can also improve the body’s immunity. This review focuses on recent advances in antiviral research on the sulfated polysaccharides from seaweeds, including carrageenan, galactan, fucoidan, alginate, ulvan, p-KG03, naviculan, and calcium spirulan. We hope that this review will provide new ideas for the development of COVID-19 therapeutics and vaccines.

## 1. Introduction

In recent years, viral infection has gradually become an important factor threatening human health and is one of the leading culprits of human death worldwide. The severe acute respiratory syndrome coronavirus 2 (SARS-CoV-2) emerged towards the end of 2019, causing COVID-19 viral pneumonia [1]. The virus is a member of the β-genus of the coronavirus family, which mainly causes infections in the pulmonary and digestive tract, and is a close relative of the SARS-CoV virus that caused the 2002–2003 atypical pneumonia outbreak [1,2,3]. It is reported that both viruses bind to the angiotensin-converting enzyme 2 (ACE2) cell receptor via a highly glycosylated spike protein (S-protein) and then enter the cell through membrane fusion or endocytosis [4,5,6,7,8]. The spike protein on the surface of the virus binds to vascular cells in the heart and kidney and epithelial cells in the lung and intestine through angiotensin-converting enzymes [9]. As binding by the spike protein is necessary for viral entry into the cell, interference with its interaction with ACE2 and prevention of viral transcription and replication is a reasonable strategy to combat SARS-CoV-2 infection [10]. However, drug development is both costly and time-consuming; thus, the discovery and development of broad-spectrum antiviral agents would be highly advantageous.

It is well known that approximately 70% of the world’s surface is covered by the oceans, giving rise to unique marine environments distinct from those on land. Algae are an important part of the marine ecosystem and are found in a wide variety of forms, from microscopic blue-green algae in plankton to kelp species that may be up to several meters in length [11]. Microalgae have traditionally been classified according to their cytological and morphological characteristics, types of reserve metabolites, cell wall components, and pigments, and include the cyanobacteria, diatoms, and Chrysophyceae [12]. Macroalgae are defined by both morphological and chemical features, especially in terms of the presence of specific pigments, and are thus divided into red, brown, and green algae [13]. Many researchers see macroalgae as an excellent opportunity to discover an inexhaustible resource of new bioactive compounds that can be used as treatments. Marine algae have long been harvested, producing large annual yields and significant economic benefits [14,15]. About 9% of the biomedical compounds in natural marine products on the market are isolated from algae [15]. These marine organisms can synthesize different types of metabolites, including polysaccharides, chlorophyll, vitamins, acetyltogenins, fatty acids, amino acids, and halogenated compounds [16,17]. Recent scientific studies have shown that algae contain a wide variety of bioactive components that have the potential for treating cancer and bacterial and viral infections, and reducing oxidative stress and inflammation, and preventing angiogenesis [18,19,20].

Marine algae are exposed to environmental stresses, including high salinity, low temperatures, high pressure, and lack of nutrients, and have adapted by the development of a wide variety of sulfated polysaccharides with diverse functions and which have found applications in both food and pharmaceuticals. As early as 1958, Gerber and colleagues showed that a polysaccharide from *Gelidium cartilagineum* (L.) *Gaillon* had some antiviral activity against the influenza B and mumps viruses, and subsequent findings have shown that polysaccharides derived from seaweeds are effective antiviral agents [21]. The polysaccharides from algae differ from their terrestrial plant counterparts in having structures that are rich in sulfated and uronic acid residues [22]. Sulfated polysaccharides have been demonstrated to have antiviral, anti-inflammatory, antioxidant, antiarteriosclerosis, and antitumor activities [23,24,25,26,27] In addition, marine sulfated polysaccharides have the advantages of low toxicity, good biocompatibility, and immunoregulatory abilities [28,29].

**Figure 1:**
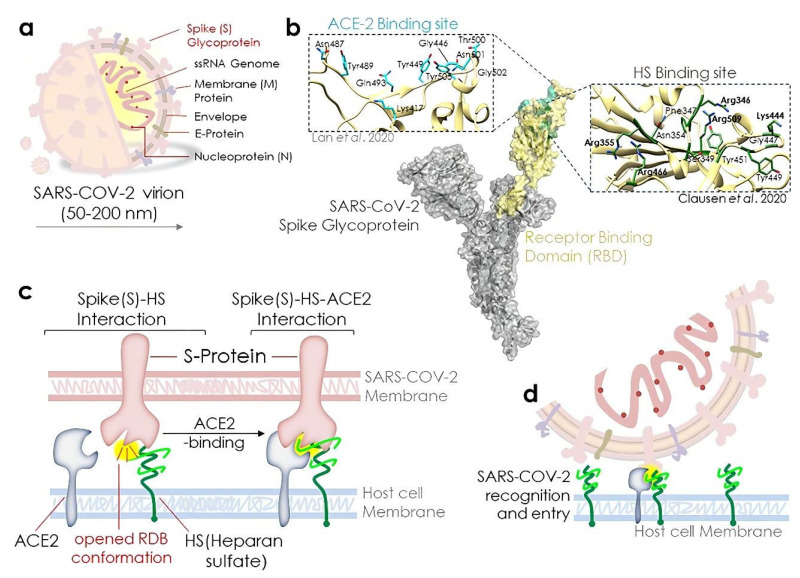
(**a**–**d**) Heparan sulfate plays an important role in the binding of the SARS-CoV-2 spike protein (S-protein) to ACE2 and related viral infections. Reprinted with permission from Ref. [34]. ©2021, Rajkumar Sigh Kalra et al., (CC BY 4.0) (for a detailed interpretation of Figure 1, the reader is referred to [34,36]).

Studies have shown that the SARS-CoV-2 spike glycoprotein (SGP) interacts with glycosaminoglycans and heparan sulfate (HS) components on the host cell membrane, possibly facilitating viral entry into the cell [30,31,32]. Surface plasmon resonance (SPR) studies have demonstrated that SGP has a high affinity for heparin binding, suggesting that heparin components may be useful targets for treating COVID-19 [4,33]. As shown in Figure 1, Kalra et al. described the important role of heparan sulfate in facilitating the opening of spike protein conformation for ACE2 binding, which can potentiate SARS-CoV-2 infection [34]. Marine sulfated polysaccharides are HS analogs and can thus simulate the effects of endogenous factors and inhibit viral interactions [35]. Therefore, marine sulfated polysaccharides may be potential candidates for preventing and treating SARS-CoV-2 infection. In this review, we summarize and analyze recent studies on the antiviral activities of the algal sulfated polysaccharides that are commonly used in industry, to provide new ideas for the development of drugs and vaccines to treat COVID-19.

## 2. Sulfated Polysaccharides from Different Seaweeds

### 2.1. Red Seaweed

#### 2.1.1. Carrageenan

Carrageenan is a soluble sulfated galactan isolated from red seaweed where it is a component of the outer cell wall and intracellular matrix, and it accounts for 30–70% of the dry weight of red algae. The galactan backbone is produced in the Golgi apparatus and is subsequently sulfated by sulfotransferases in the cell wall [37]. Commercially, red seaweed is considered more valuable than brown and green algae. Furthermore, the kappa-(κ-), iota-(ι-), and lambda-(λ-) isoforms are most commonly used in industry. These differ in the position and numbers of sulfate groups attached to the hexose scaffold, with the κ, ι, and λ forms containing one, two, and three anionic sulfate ester moieties, respectively, on each disaccharide repeat [38]. The degree of sulfation of the κ-, ι, and λ isoforms is 25–30%, 28–30%, and 32–39%, respectively [39]. The sol-gel transition, chemical cross-linking, mechanical strength, and biological properties vary with the structural changes of carrageenan. Due to its unique properties, carrageenan is mainly used in industries, such as food, cosmetics, printing, textile formulations, and pharmaceuticals [40]. Significantly, a higher degree of sulfation in carrageenan does not necessarily correspond with higher antiviral activity [41], and it appears that both the position and density of the sulfate moieties on the backbone influence the molecule’s antiviral capability [42]. This suggests that carrageenan’s antiviral activity is not entirely dependent on the sulfate content. In addition, carrageenan is the most studied sulfated polysaccharide in human clinical trials for use against various viral diseases [43].

##### Kappa-(κ-)carrageenan

Kappa-(κ-)carrageenan inhibits viral replication both through blocking adsorption to the surface and inhibition of protein expression. Low-molecular-weight κ-carrageenan shows a better performance in these aspects [44,45]. Shao et al. investigated the molecular mechanism by which k-carrageenan protects cells from invasion by H1N2009 influenza (SW731) virus [44]. They treated MDCK cells with κ-carrageenan, observing significant inhibition of SW731 influenza virus replication resulting from interference with viral adsorption and protein expression [44]. Remarkably, low-molecular-weight κ-carrageenan has better antiviral activity because of its better tissue penetration. Wang and colleagues found that 2-kDa-κ-carrageenan (CO-1) prevented the replication of the influenza A (H1N1) virus in MDCK cells more effectively than the 3 and 5 kDa forms (CO-2 and CO-3, respectively), with IC50 values of 32.1, 239, and 519 µg/mL for the 3 isoforms, respectively [45]. Given these results, the authors recommend the use of low-molecular-weight carrageenan oligosaccharides for influenza treatment as an alternative strategy [45]. Schütz et al. showed that both nasal and oral sprays containing κ-carrageenan inhibited SARS-CoV-2 replication in human airway epithelial cells [46]. Furthermore, κ-carrageenan also showed significant inhibitory effects on HSV-2 and HPV16, with IC50 values of 1.6 and 0.044 µg/mL, respectively [40,47].

##### Lambda-(λ-)carrageenan

Lambda-(λ-)carrageenan inhibits viral activity by inhibiting viral internalization through targeting attachment cell surface receptors and binding to viral envelope proteins [48,49,50]. Luo et al. found that λ-carrageenan P32 screened from different molecular weights (4–350 kDa) carrageenans had the highest inhibitory effect on RABV infection, which was consistent with the low molecular weight (4 kDa), high solubility, and high stability of closely related P32 [48]. These results suggested that λ-carrageenan P32 was a promising drug to inhibit RABV infection by preventing virus internalization and glycoprotein-mediated cell fusion [48]. In mice, the use of λ-carrageenan nasal drops not only reduced weight loss resulting from influenza viral infection but also prevented infection-related death in a majority of the mice [49]. In addition, λ-carrageenan was also effective against SARS-CoV-2. The EC50 of λ-carrageenan was 0.9 ± 1.1 μg/mL and the principal mechanism involved λ-carrageenan targeting viral attachment to cell surface receptors and subsequent prevention of viral entry [49]. λ-carrageenan extracted from the red alga *Gigantina skotsbergii* was found to be effective in preventing infection by equid herpesvirus 3(EHV3), bovine herpesvirus 1 (BoHV-1), and suid herpesvirus-1 (SuHV-1), most likely because the compound binds to the envelope glycoprotein of the virus, preventing viral attachment to the cell surface receptor [50]. In addition, λ-carrageenan also had a significant inhibitory effect on DENV-3, with an EC50 of 0.14 µg/mL [51]. λ-carrageenan polysaccharide induces the synthesis of interferon and has biological effects on the immune response. Studies have shown that microwave degradation of λ-carrageenan from *Chondrus ocellatus* can inhibit tumor growth, enhance interferon activity, and enhance lymphocyte multiplication [52]. However, it has been reported that λ-carrageenan can induce enteritis in rats after long-term oral administration [53]. Furthermore, these sulfated polysaccharides can be used in the manufacture of carbohydrate-based conjugate vaccines to achieve the desired immunogenicity and potency. Luo et al. proved that λ-carrageenan has increased the efficacy of ovalbumin-based prophylactic and therapeutic cancer vaccines [54].

##### Iota-(ι-)carrageenan

Iota-(ι-)carrageenan’s antiviral activity against a variety of viruses, especially respiratory viruses, has been well documented [55,56,57,58]. Some scholars have proposed to improve the therapeutic effect by combining carrageenan with other clinically common antiviral drugs. A study using a combination of ι-carrageenan and oseltamivir showed that this combination significantly improved the survival rate of infection with the H1N1 virus compared with a single therapy [57]. Ludwig et al. found that patients treated with ι-carrageenan recovered more quickly than those in the placebo group, with a duration of 11.6 days compared with 13.7 days in the placebo group. In addition, the ι-carrageenan group showed significantly faster remission of symptoms than the placebo group, with lower viral loads in the nasal cavity [59]. ι-carrageenan from *Euchema spinosum* was able to neutralize the SARS-CoV-2 Spike pseudotyped lentivirus (SSPL) in a concentration-dependent manner at an MOI of 0.1 and an IC50 of 2.6 µg/mL [60]. In addition, different forms of administration of ι-carrageenan can play a significant role in its antiviral activity. Morokutti et al. found that ι-carrageenan in lozenge form significantly reduced the amount of SARS-CoV-2 virus in saliva, thus limiting interpersonal viral transmission and the transfer of the virus to the lower respiratory tract [61]. In addition, ι-carrageenan can be given directly to infected patients to treat COVID-19 with ivermectin via nasal spray and oral antivirals. The number of people diagnosed with COVID-19 in the treatment group was 3.4%, which was significantly lower than 21.4% in the control group (*p* = 0.0001) [62]. The Xylitol^®^ nasal spray containing ι-carrageenan has been found to prevent SARS-CoV-2 infection in vitro, with an IC50 < 6.0 µg/mL [63]. Graf et al. developed a nasal spray formulation containing 0.05% xylimeta-zoline hydrochloride and 0.12% ι-carrageenan. The formulation was reported to be effective in relieving nasal congestion symptoms while also providing antiviral protection to the respiratory mucosa [64]. Hassanzadeh et al. found that ι-carrageenan can significantly inhibit SARS-CoV-2 in vitro, an effect caused by the effect of positively charged regions on the glycoprotein envelope and protein aggregation in host cells on the surface [65].

Although the three types of carrageenan, namely, κ-, ι-, and λ-carrageenan, showed antiviral action against SARS-CoV-2, including the alpha, beta, gamma, and delta variants of concern [46,49,60], ι-carrageenan showed the strongest antiviral activity with an IC50 value approximately ~1 log-stage lower than either λ-or κ-carrageenan [66]. Therefore, ι-carrageenan is a potential respiratory virus inhibitor that can be used to prevent and treat SARS-CoV-2 infection, irrespective of the viral variant.

In addition, the combined use of antiviral drugs often has a multiplier effect. Morokutti-Kurz et al. showed that carrageenan and Zanamivir act synergistically against several influenza A virus strains (H1N1(09)pdm, H3N2, H5N1, H7N7) in vitro; therefore, by acting synergistically, they can provide a broader spectrum of anti-influenza activity [58]. When using ι- and k-carrageenan at the same time, the physical interaction of carrageenan with the virus did not interfere with the inhibitory effect of zanamivir, and the spray effect was increased [58]. Xylimidazolines have been used for over 50 years to relieve vasoconstriction and acute nasal edema. Graf et al combine this vasoconstrictor and ι-carrageenan in a scientific formulation. The experimental results show that ι-carrageenan does not reduce the efficacy and safety of the drug, and the antiviral effect of iota-carrageenan is also not affected [64]. Therefore, the most successful antiviral formulation of carrageenan may be the recently developed nasal spray formulation for use against rhinoviruses and SARS-CoV-2 [63,64].

#### 2.1.2. Galactan

Sulfated galactans are the principal extracellular polysaccharides found in red seaweed. With few exceptions, they consist mainly of linear chains of galactose. These polysaccharides show good antiviral activity against HSV, DENV, HIV, and HAV [67,68,69,70]. Galactan from the red alga *Agardhiella tenera* has been shown to inhibit HIV-1 and HIV-2 infection by preventing the interaction between HIV gp120 and the CD4 + T cell receptor [69,71]. Similarly, 12.5 μg/mL galactan isolated from *Schizymenia binderi* was found to inhibit HIV replication in vitro, and block the replication of HSV-1 in Vero cells [72]. Matsuhiro et al. found that *Schizymenia binderi* galactan showed strongly selective antiviral activity against HSV-1 and HSV-2, with EC50 values of 0.76 and 0.63 μg/mL, respectively [73]. Similarly, 3 galactan (F1, F2, and F3) isolated from Callophyllis variegate are effective inhibitors of HSV-1 and HSV-2, with IC50 values ranging from 0.16 to 2.19 μg/mL, and are effective against DENV-2 with IC50s in the range of 0.10–0.41 μg/mL [74]. The galactan (C2S-3) extracted from *Cryptonemia crenulata* can inhibit the proliferation of DENV-2 in Vero cell lines [75]. The result of the experiment showed that C2S-3 blocked the initial binding of the virus to cells and its subsequent penetration, preventing DENV-2 from RNA replication and other biomacromolecule synthesis functions in host cells. Moreover, compared with heparin, C2S-3 was more effective as an antiviral against various DENV-2 strains [75]. Therefore, galactan is a very promising antiviral drug.

### 2.2. Brown Seaweed

#### 2.2.1. Fucoidan

Fucoidan is an intercellular or mucilage matrix component of brown seaweed, accounting for approximately 5–20% of the dry weight of the plant [76,77]. Fucoidan is documented to be effective against a wide variety of viruses, including HIV, HSV, and SARS-CoV-2, and numerous other RNA and DNA viruses [78,79,80,81,82,83,84]. Dinesh et al. extracted fucoidan (CFF, FF1, and FF2) from *Sargassum swartzii*, observing that the FF2 fraction was effective against HIV-1 at concentrations between 1.56 and 6.25 μg/mL, shown by significant reductions in the p24 antigen levels (95.6 ± 1.1%) and reverse transcriptase (78.9 ± 1.43%) at a concentration of 25 µg/mL [81]. Fucoidans isolated from *Dictyota mertensii, Lobophora variegate, Fucusvesiculosus,* and *Spatoglossum schroederi* were found to inhibit HIV reverse transcriptases, thus preventing infection; it was further observed that the antiviral action was positively associated with the numbers of sulfate moieties on the compound [85]. Lee et al. demonstrated that the fucoidan extracted from Mekabu and *Sargassum trichophyllum* significantly inhibited HSV-1, HSV-2, H5N3, and influenza A viral infection together with enhancing the immune function [86]. High-molecular-weight fucoidan (KW) from the brown alga *Kelmanella crassifolia* was shown to bind and block influenza A virus neuraminidase activity, inhibiting the release of viral particles. Fucoidan was also found to block EGFR and subsequent activation of downstream PI3K/Akt and NF-κB signaling [87]. In addition, fucoidan also inhibits NDV La Sota infection (IS_50_ > 2000), significantly reducing the number of syncytia (inhibition rate of 70%), suggesting specific binding of fucoidan to the F0 protein [88]. Fucoidan is considered a possible candidate for treating COVID-19 as it has significant antiviral activity [89]. Recovery of the mitochondrial membrane potential Δψm was observed in the PBMCs of patients after recovery from COVID-19, showing that fucoidan has strong antioxidant activity and can restore cellular homeostasis [90,91,92]. RPI-27 extracted from Saccharina japonica is a high-molecular-weight fucoidan similar in structure to glycosaminoglycans on the surfaces of host cells [93]. This could provide opportunities for binding the S protein of SARS-CoV-2, resulting in competitive inhibition with the virus, with an EC50 value of 8.3 ± 4.6 μg/mL [93]. Yuguchi et al. reported that the fucoidan derived from *Sargassum crassifolium* and *Padina australis* showed similar anti-HIV activity by blocking an early step in HIV entry into target cells [94].

Fucoidan has a variety of immunomodulatory effects, such as stimulating the production of NK (natural killer) cells, promoting cell development and other functions of dendritic cells. In addition, it enhances Th1-type immune responses by producing antibodies against specific antigenic determinants and generating memory T cells against specific viruses [95]. Sulfated polysaccharides could provide an important approach to designing therapeutic vaccines based on their desired physicochemical properties and easily modifiable structural features. Fucoidan is reported to have the best adjuvant quality for future vaccine production and can elicit strong cell-mediated and humoral immune responses [96].

#### 2.2.2. Alginate

Alginate is a soluble acidic polysaccharide found in the cell walls of brown seaweed, especially *Macrocystis pyrifera, Laminaria hyperborea*, and *Ascophyllum nodosum*, amongst others [97]. Alginate is a linear polymer formed by 1,4-linked β-D-mannuronic acid and 1,4 α-L-guluronic acid moieties assembled in blocks [98]. The compound has both antiviral and immunomodulatory activities [99,100,101,102,103]. Serrano-Aroca et al. summarized and analyzed the effects of biomaterials constructed of alginate on 17 viruses, finding that these materials were essentially non-toxic and effective against a variety of viruses [104]. In vivo results showed that oral administration of marine polysaccharide drug 911 reduced viral infection and the plasma RNA copy number. In addition, the introduction of 911 has a protective effect on CD4 cells [105,106,107]. Furthermore, the inhibitory effect of 911 on HIV-1 is dose dependent with low toxicity. Moreover, it can also inhibit HBV viral replication by inhibiting DNA replication [108].

##### Polymannuroguluronate

Polymannuroguluronate (PMG) is a common low-molecular-weight alginate. Polymannuroguluronate sulfate (PMGS) is capable of inactivating HPV particles and of blocking virus capsid L1 protein binding, and downregulating the levels of the E6 and E7 viral oncogenic proteins [109]. In addition, sulfated polymannuronate (SPMG) inhibits the interaction between the HIV-1 gp120 protein and the CD4 + T lymphocyte receptor, thus preventing entry of the virus into the lymphocyte [110]. In addition, Miao et al. suggested that the interaction between SPMG and the CD4 + T lymphocyte may provide a mechanistic explanation for the immunoenhancement and anti-AIDS activity of SPMG in HIV-infected individuals [111]. Therefore, PMGS deserves further study as a novel candidate for the prevention of HPV infection, treatment of genital warts or cervical cancer, and HIV infection.

##### Polyguluronate

Polyguluronate (PG) is another low-molecular-weight alginate. Polyguluronate sulfate (PGS) significantly reduces the levels of HBsAg (51.8%) and HBeAg (36.2%), showing dose- and time-dependent inhibitory effects [112]. PGS likely binds to HepG2.2.15 cells, upregulating the NF-κB and RAF/MEK/ERK pathways to promote interferon-β production and thus interfering with HBV transcription and exerting an anti-HBV effect [112]. In addition, PGS can significantly reduce oxidative stress induced by H_2_O_2_ and improve the survival rate of HepG2 hepatocytes due to its strong antioxidant activity [113]. Therefore, PGS, as a new anti-HBV drug designed to regulate the host’s natural immune system, deserves further study.

### 2.3. Green Seaweed

#### Ulvan

Ulvan is the most common polysaccharide in the cell walls of green seaweed, making up to 8–29% of the algal dry weight [114]. Both in vitro and in vivo investigations have shown that ulvan has anticoagulant, antibacterial, antiviral, and immunomodulatory activities [115,116,117,118,119]. Several low-molecular-weight ulvan isoforms (ULVAN-F1, ULVAN-F2, and ULVAN-F3) were purified from *Ulva pertusa*, which were found to be effective in preventing the infection and replication of vesicular stomatis virus [120]. The antiviral activity of ulvan is not, however, consistently related to its molecular weight. Sun et al. found that both ulvan purified from *Ulva pertusa* and its low-molecular-weight degradation product, LUPP-3, had strong inhibitory effects [121]. In addition, ulvan from *Ulva intestinalis* effectively suppressed measles virus (MeV) by reducing the formation of syncytia, with an IC50 of 3.6 μg/mL [122], and ulvan from *Ulva clathrata* can effectively inhibit NDV by inhibiting cell-to-cell fusion through the direct action of F0 protein, with an IC50 of 0.1 μg/mL [123]. The results showed that ulvan inhibited syncytium formation only before F protein cleavage. Therefore, the antiviral activity of ulvan relies on interaction with intact F0 protein rather than cleaved mature F protein [123]. The antiviral effect of SU1F1 is mainly via inhibition of DNA replication and transcription while downregulating HSV protein synthesis [124]. The polysaccharide extract containing ulvan blocks the adsorption of JEV (Japanese encephalitis virus) and inhibits the entry of the virus into the host cell. In addition, they effectively reduce the production of proinflammatory cytokines [125]. In addition to antiviral activity, ulvan also has certain immunomodulatory activity. Ulva extract from *Ulva armoricana* can induce the release of proinflammatory cytokines by activating avian heterophile and monocytes in vitro, ultimately enhancing the innate immune system of chickens [126]. Ulvan from green seaweed still warrants further research, although studies on its isoforms suggest that it is not more effective than polysaccharides from red and brown seaweed.

### 2.4. Microalgae

#### 2.4.1. p-KG03

p-KG03 is a homogeneous polysaccharide derived from *Gyrodiniumimpudicum* and complexed by galactose with uronic and sulfonic acid groups [67]. p-KG03 extracted from the dinoflagellate *Gyrodinium impudicum* is the first marine compound (EC50 = 26.9 μg/mL) reported to significantly inhibit encephalomycarditis RNA virus (EMCV) infection in vitro [127]. Kim et al. also observed that p-KG03 was effective against IAV infection [128]. Further investigation of the mechanism showed that p-KG03 had the greatest inhibitory effect on IAV replication within six hours, suggesting that the compound targeted the steps of virus adsorption and internalization [128]. Therefore, the potential antiviral activity of p-KG03 indicates that it is a promising candidate for development as an antiviral drug.

#### 2.4.2. Naviculan

Naviculan is derived from the diatom *Navicula directa*. Lee and colleagues reported that naviculan reduced virus infection by inhibiting the binding and internalization of HSV-1, HSV-2, HIV, and INF A, with IC50 values in the 7.4–170 μg/mL range [129].

#### 2.4.3. Calcium Spirulan

Calcium spirulan is obtained from the alga *Arthrospira platensis*. Due to the chelation of calcium ions with the sulfate groups on the polysaccharide, it has high antiviral activity against coated viruses [130,131,132,133]. Hayashi and colleagues found that calcium spirulan (Ca-SP) from *Spirulina platensis* is an inhibitor of several viruses. It can inhibit replication and infiltration of HSV-1, HCMV, MeV, MUV, INF A, and HIV-1, with EC50 values in the 0.92–23 μg/mL range [134].

## 3. Conclusions and Future Outlooks

Sulfated polysaccharides are important bioactive substances in marine algae, and have various biological activities, of which their antiviral actions are especially attractive. In addition, their high yields, low production costs, broad-spectrum antiviral activities, and unique antiviral mechanisms suggest that sulfated polysaccharides from seaweeds are promising antiviral drugs. It should be noted that the source of marine polysaccharides is not limited to seaweed but also includes a large number of marine animals and marine microorganisms, such as *Thais clavigera* [135], *Sepia pharaonis* [136], sea cucumbers [137], *Haliotis discus hannai* [138], and *Celtodoryxgirardae* [139].

In Table 1, we summarize recent studies on the effects of sulfated polysaccharides from different marine algae on DNA and RNA viruses. From these studies, we may conclude that: (1) there are more kinds of sulfated polysaccharides from red and brown algae, and there are more studies on the antiviral activities of sulfated polysaccharides from different seaweed; (2) sulfated polysaccharides from different seaweeds have significant inhibitory actions against various DNA and RNA viruses; (3) the inhibitory activities of sulfated polysaccharides from different seaweeds towards the same virus differ; and (4) the antiviral activities of the sulfated polysaccharides vary according to the type of virus and the type of host cell. Therefore, the antiviral effect of sulfated polysaccharides is not only related to the type and source of sulfated polysaccharides but also closely related to the type of host cell, virus type, and other factors. This suggests that we should not only focus on the antiviral drug polysaccharide itself but also explore more factors that affect the antiviral effect when researching antiviral polysaccharides. Furthermore, more detailed research into the antiviral mechanisms of sulfated polysaccharides is required. Sulfated polysaccharides have complex antiviral mechanisms that essentially include two aspects: (1) inhibition of virus activity and (2) enhancement of the host immune response to the virus.

**Figure 2:**
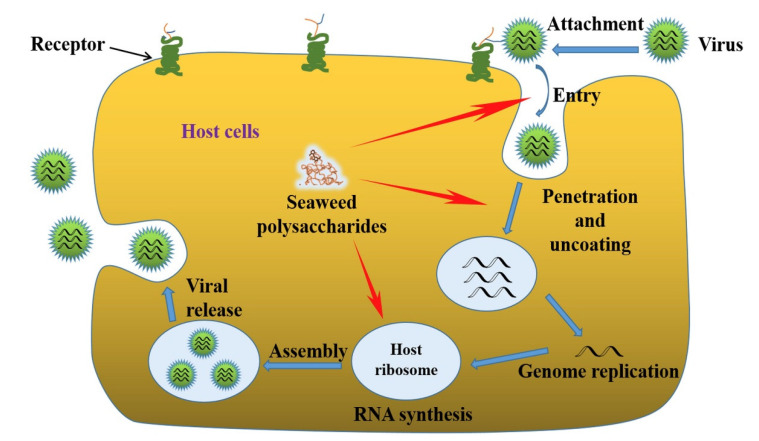
Viral infection and the antiviral phase of seaweed polysaccharides. Reused with permission, license number: 5265040871103 Adapted with permission from Ref. [140]. ©2017, Elsevier Ltd. All rights reserved.

As shown in Figure 2, although the life cycle of a virus varies from species to species, it includes six basic stages: attachment, penetration, uncoating, replication, assembly, and release. The initial stage of virus entry into cells is by attaching to the cell surface through receptors (heparan sulfated proteoglycan), modifying their surface, or by electrostatic means. Seaweed polysaccharides can target the viral attachment stage by directly interacting with virions or by mimicking the binding of virus-associated proteins to the corresponding receptors The internalization process of viruses usually involves the following three steps: endocytic uptake, vesicular transport, and then delivery to the endosomes and other intracellular organelles. Antiviral strategies targeting the viral penetration and uncoating stages typically interfere with the release of endosomal DNA and RNA by blocking structural changes in viral glycoproteins. In addition, seaweed polysaccharides can inhibit viral transcription and replication through direct interference with viral replication enzymes or inhibition on other intracellular targets [140]. Marine polysaccharides, especially sulfated polysaccharides from marine algae, appear to block viral infection by inhibition of one or more of these stages, with the specific mechanisms dependent on the structure of the individual polysaccharide [140,141,142,143,144].

**Figure 3:**
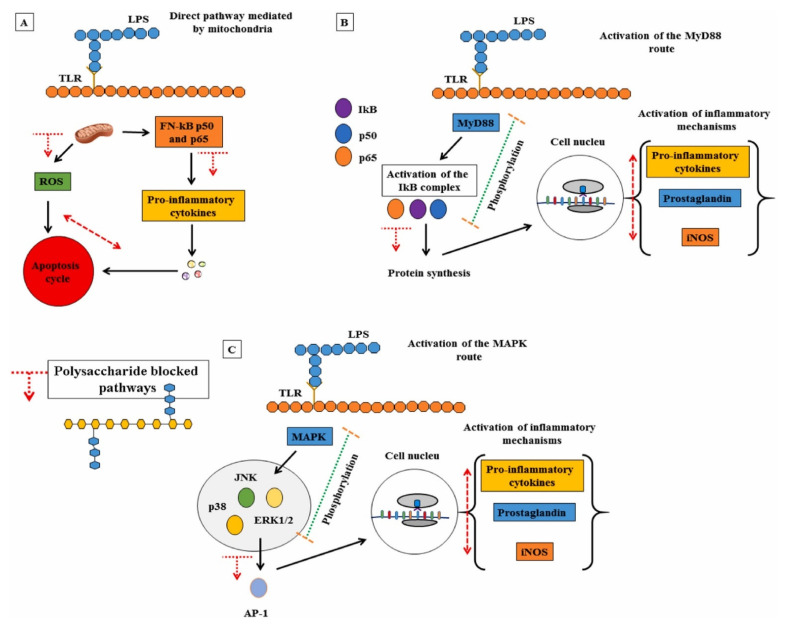
Major inflammatory pathways mediated by the immune system and polysaccharide signaling mechanisms that may contribute to immunosuppressive pro-inflammatory production pathways. (**A**) Direct route mediated by mitochondria; (**B**) MyD88 protein signaling pathway; (**C**) MAPK protein signaling pathway. Reprinted with permission from Ref. [145]. ©2021, Elsevier Ltd. All rights reserved. (for a detailed interpretation of Figure 3, the reader is referred to [145,146]).

Since sulfated polysaccharides are an abundant source of polyanions, they can stimulate the production of interferon by host cells, thereby promoting the immune response to viral infection. Furthermore, sulfated polysaccharides can lead to effective immune activation and modification of cell signaling mechanisms, thereby eliciting higher antiviral responses in cells. Barbosa et al. summarized several pathways of polysaccharides that activate immunosuppression, and all the mechanisms described can be seen in Figure 3, including Figure 3A direct route mediated by mitochondria, Figure 3B MyD88 protein signaling pathway, and Figure 3C MAPK protein signaling pathway [145]. These studies should be focused on the effects of polysaccharides for modulating the immune systems, especially via cytokines (TNF-α and IL-6) release, increased phagocytosis of macrophages, production of nitrous oxide (NO), reactive oxygen species (ROS) formation, and signaling pathway activation (e.g., toll-like 4, type A hijacker receptor, NF-κB, and glucan receptor) [145,146,147,148]. In addition, sulfated polysaccharides can also enhance the immune response of the host by stimulating the production of immune factors to indirectly block viral replication and facilitate viral clearance [140,149]. Therefore, sulfated polysaccharides are suitable interferon inducers, natural immunomodulatory drugs, or dietary supplements for the treatment of COVID-19 [150].

Vaccination can effectively reduce deaths from infectious diseases and promote the anticipation of life. Due to the nature and mutation of host strains, the development of coronavirus vaccines is critical. Adjuvants are biologically active substances added to vaccines to promote immune responses with vaccine antigens [151]. Long-term immune memory and protection of the immune system can be achieved by adding adjuvants to enhance vaccine efficacy. Studies have shown that sulfated polysaccharides have strong immune-stimulating activity and are suitable as adjuvants for various vaccines [152,153]. Therefore, sulfated polysaccharides can be used as a potential adjuvant candidate for the production of SARS-CoV-2 antiviral vaccines [153]. However, further in vivo studies and validations are needed to obtain an effective vaccine against the coronavirus.

Oxygen-free radicals and nitric oxide can cause oxidative tissue damage. Additional evidence suggests that patients affected by either RNA virus may experience chronic oxidative stress. Therefore, antioxidants are also an antiviral drug that cannot be ignored [154]. SARS-CoV-2 is a positive-stranded single-stranded RNA virus with a high mutation rate, which causes it to escape host immunity and currently exhibits drug resistance [155]. Sulfated polysaccharides are good sources of antioxidants for their effective scavenging and chelating potential in various applications [156,157]. The structure of sulfated polysaccharides has so far shown remarkable antioxidant activity and reduced oxidative stress in various diseases [158,159].

The antiviral activity of marine sulfated polysaccharides is largely dependent on their structures, specifically, the degree and type of sulfation, molecular weight, and monosaccharide compositions, together with other structural characteristics, such as the three-dimensional structure and hydrogen bonds [160,161]. Polysaccharides are usually extracted and purified from marine algae, and these procedures may be complicated by the diversity of structures and characteristics of these molecules [162]. Water extraction and alcohol precipitation are commonly used to extract and purify marine polysaccharides, which is different from the extraction method of terrestrial plants using toxic organic reagents. Seaweed polysaccharides cannot be digested by digestive enzymes in the human body. However, these biopolymers can effectively increase the activity of beneficial bacteria in the gut [100]. The physicochemical and mechanical properties of sulfated polysaccharides can be easily modified, increasing their application in the pharmaceutical industry [163]. Chemical synthesis has also been put into practice as an effective alternative to obtaining pure marine oligosaccharides with specific structures [140]. At present, the commonly used methods for sulfation include the Nagasawa method, Wolfrom method, and SO_3_-pyridine method [164,165,166]. Although the chemical synthesis and purification of polysaccharides are still difficult, the further development of chemical synthesis will surely provide new possible routes for the synthesis of those marine polysaccharides with well-defined structures.

Significantly, most of the current pharmacological investigations into the effects of polysaccharides are restricted to preclinical studies. For example, many reported pharmacological studies are limited to in vitro studies of single host cells. However, clinical evaluation is key to drug development, and such studies will provide a foundation for the clinical application of these potential antiviral drugs in the future. In addition, the combined use of a variety of different sulfated polysaccharides, the combined use of polysaccharides and common antiviral drugs, and different dosage forms of the same drug or different modes of administration will provide useful references to the clinical application of antiviral drugs. Furthermore, the rationality and safety of the clinical compatibility of related drugs need further research. Research into the antiviral actions of sulfated polysaccharides derived from marine algae on SARS-CoV-2 has resulted in much new information. These studies also provide strong support for the development and application of algae-derived drugs in the future.

## Figures and Tables

**Table 1:** Advances in research on the antiviral activity of sulfated polysaccharides from seaweeds.

Seaweed Name (Source)	Polysaccharide Name	Virus	Group	Cell Lines	Efficacy (µg/mL)	Reference
Red seaweed	κ-carrageenan	HPV16	DNA	Hela	IC50 = 0.044 µg/mL	[40]
		H1NI	RNA	MDCK	IC50 = 32.1 µg/mL	[45]
		HSV-1	DNA	Vero	IC50 = 1.9 µg/mL	[47]
		HSV-2	RNA	Vero	IC50 = 1.6 µg/mL	[47]
	λ-carrageenan	RABV	RNA	NA	IC50 = 22.1 µg/mL	[48]
		RABV	RNA	BSR	IC50 = 57.7 µg/mL	[48]
		RABV	RNA	SK-N-SH	IC50 = 19.93 µg/mL	[48]
		SARS-CoV-2	RNA	Vero	IC50 = 0.9 ± 1.1 µg/mL	[49]
		DENV-2	RNA	HepG2	EC50 = 0.22 µg/mL	[51]
		DENV-2	RNA	Vero	EC50 = 0.15 µg/mL	[51]
		DENV-3	RNA	HepG2	EC50 = 0.14 µg/mL	[51]
	ι-carrageenan	DENV-2	RNA	Vero	EC50 = 0.4 µg/mL	[55]
		H1N1	RNA	MDCK	IC50 = 0.39 µg/mL	[58]
		H3N2	RNA	MDCK	IC50 = 0.92 µg/mL	[58]
		H5N1	RNA	MDCK	IC50 = 10.14 µg/mL	[58]
		SARS-CoV-2	RNA	Vero	IC50 = 0.046 µg/mL	[60]
	Galactan	HIV-2	RNA	Vero	EC50 = 4.7 µg/mL	[70]
		HSV-1	DNA	Vero	IC50 = 4.1 µg/mL	[72]
		DENV-2	RNA	Vero	EC50 ≈ 1 µg/mL	[75]
Brown seaweed	Fucoidan	HIV-1	RNA	CD4	IC50 = 0.33–0.7 µg/mL	[84]
		H3N2	RNA	MDCK	IC50 < 6.5 µg/mL	[87]
		NDV	RNA	Vero	IC50 = 0.75 ± 1.6 µg/mL	[88]
		SARS-CoV-2	RNA	Vero	EC50 = 8.3 ± 4.6 µg/mL	[93]
	PMGS	HPV	DNA	Hela	IC50 = 2.8 µg/mL	[109]
		HIV-1	RNA	CD4	IC50 = 30 µg/mL	[111]
	PGS	HBV	DNA	HepG2	EC50 ≈ 250 µg/mL	[112]
Green seaweed	Ulvan	Measles	RNA	Vero	IC50 = 3.6 µg/mL	[122]
		NDV	RNA	Vero	IC50 = 0.1 µg/mL	[123]
		HSV	DNA	HEp-2	IC50 = 28.25 µg/mL	[124]
Microalgae	p-KG03	EMCV	RNA	MT-4	EC50 = 26.9 µg/mL	[127]
		H1N1	RNA	MDCK	EC50 = 0.48 ± 0.23 µg/mL	[128]
	Naviculan	HSV-2	DNA	CD4	IC50 = 7.4 µg/mL	[129]
	Calcium Spirulan	HSV-1	DNA	Hela	EC50 = 0.92 µg/mL	[134]

## Data Availability

Data is contained within the article.
